# A randomised trial of pre-exercise meal composition on performance and muscle damage in well-trained basketball players

**DOI:** 10.1186/1550-2783-11-33

**Published:** 2014-06-25

**Authors:** Hannah Lonika Gentle, Thomas Darrell Love, Anna Susan Howe, Katherine Elizabeth Black

**Affiliations:** 1 Department of Human Nutrition, University of Otago, P.O. Box 56, 9054 Dunedin, New Zealand; 2 Applied Sports Technology Exercise and Medicine Research Centre Sport & Exercise Science College of Engineering, Swansea University, Room 955 Talbot Building, SA2 8PP Swansea, Wales

**Keywords:** Creatine kinase, Protein, Pre-exercise, Macronutrient

## Abstract

**Background:**

Attenuating muscle damage is important to subsequent sports performance. It is possible that pre-exercise protein intake could influence markers of muscle damage and benefit performance, however, published research provides conflicting results. At present no study has investigated protein and carbohydrate (PRO/CHO) co-ingestion solely pre-exercise, nor prior to basketball-specific exercise.

The purpose of this study was to answer the research question; would pre-exercise protein intake enhance performance or attenuate muscle damage during a basketball simulation test?

**Methods:**

Ten well-trained male basketball players consumed either carbohydrate (1 g · kg^−1^ body mass) with protein (1 g · kg^−1^ body mass), or carbohydrate alone (2 g · kg^−1^ body mass) in a randomised cross- over design, 90 minutes before completing an 87-minute exercise protocol.

**Results:**

The rise in creatine kinase (CK) from baseline to post-exercise was attenuated following PRO/CHO (56 ± 13U · L^−1^) compared to carbohydrate (100 ± 10 U · L^−1^), (p = 0.018). Blood glucose was also higher during and post-exercise following PRO/CHO (p < 0.050), as was free throw shooting accuracy in the fourth quarter (p = 0.027). Nausea during (p = 0.007) and post-(p = 0.039) exercise increased following PRO/CHO, as did cortisol post-exercise (p = 0.038).

**Conclusions:**

Results suggest that in well-trained basketball players, pre-exercise PRO/CHO may attenuate the rise in CK, indicative of a decrease in muscle damage during exercise. However, unfamiliarity with the protein amount provided may have increased nausea during exercise, and this may have limited the ability to see an improvement in more performance measures.

## Introduction

Basketball game play has been shown to elevate plasma creatine kinase (CK) immediately post-exercise [1]. Attenuating these increases in CK may assist performance towards the end of a game or improve recovery prior to the next training session [2-6].

Co-ingestion of protein and carbohydrate (PRO/CHO) before and during exercise has been shown to inhibit muscle protein degradation during and post-exercise during resistance type exercise [7,8], and attenuates indirect markers of muscle damage such as CK [9]. However, not all studies have found such promising results, with reports of no significant differences in muscle soreness, CK or myoglobin levels post-exercise following the ingestion of PRO/CHO compared to carbohydrate alone or water [10-14].

The discrepancies in results could be due to differences in training status, age and sex, as well as the exercise protocols used to induce muscle damage, the indirect markers chosen to estimate the extent of such damage, and the amount and timing of PRO/CHO consumption. Previous studies have investigated the effect of PRO/CHO ingestion on performance, markers of muscle damage, hormonal interactions, and substrate metabolism, and have provided protein at multiple stages throughout study protocols [10,12,15].

At present, there are no known studies which have focused solely on pre-exercise PRO/CHO co-ingestion, and therefore it is difficult to determine the ingestion time-point responsible for the beneficial effects. Furthermore, few previous studies have investigated PRO/CHO co-ingestion and the effect on performance in a valid team-sport setting [12]. The Basketball Exercise Simulation Test (BEST) [16], constructed using time-motion analysis of actual basketball games [17] provides the most valid form of standardised exercise protocol for basketball players.

The present study aimed to investigate the effects of iso-energetic PRO/CHO co-ingestion in comparison to carbohydrate alone on performance and markers of muscle damage in basketball players, using the BEST protocol to simulate game play.

## Methods

### Participants

Ten well-trained male basketball players (mean ± SD; age: 22 ± 2 years, height: 183.9 ± 7.5 cm, body mass: 81.8 ± 10.9 kg, body fat percentage: 9.5 ± 2.7%) volunteered and provided written informed consent to participate in this study, which was approved by the University of Otago Human Ethics Committee. Training load at the time of testing consisted of two 120-minute practices and one game (of four 10-minute quarters) per week.

### Experimental design

Participants attended three testing sessions. The first was for familiarisation with the exercise protocol, to obtain baseline body mass, and to determine ad libitum fluid (water) intake during exercise. For the two intervention sessions, following an overnight fast, participants were randomly assigned to consume either the PRO/CHO or carbohydrate meal (CHO). Ninety minutes following meal consumption, participants performed an 87-minute basketball game simulation test where sprint time, jump height, and free throw success rate were recorded. Fluid intake was individualised to match ad libitum fluid intake rate (mL · min^−1^) measured during the familiarisation session. Vene-puncture and ear-prick blood samples, saliva samples and urine samples were taken throughout the study protocol. Questionnaires were also completed, inquiring about gastrointestinal (GI) discomfort [18], muscle soreness (MS) [19] and ratings of perceived exertion (RPE) [20]. Participants were asked to keep a food diary 24-hours prior to the first intervention, and to replicate this intake before the alternate intervention. Additionally, participants were asked to refrain from strenuous exercise, protein-containing supplements, anti-inflammatory medication, pain killers and alcohol consumption in the 24-hours prior to testing.

### Exercise protocol

All exercise testing was performed on one half of a regulation International Basketball Federation basketball court. The exercise protocol consisted of; four 10-minute quarters of “game play” represented by circuits of the BEST [16], along with free throws, suicide runs [21], and rest periods, which were combined in such a way as to represent a basketball game.

### Familiarisation testing

**Figure 1:**
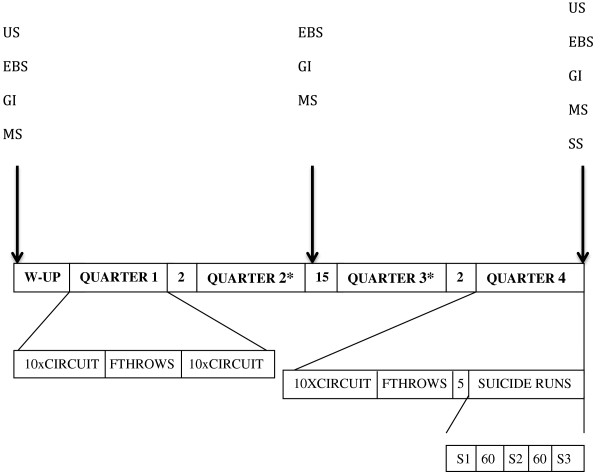
Construct of the full (87-minute) exercise protocol.

A standardised 7-minute warm-up was performed, consisting of jogging, lay-ups, running, dribbling and stretching. Participants then performed four consecutive free throws. This was followed by familiarisation with the BEST, which involved talking participants through the various movement patterns (sprinting, decelerating, sidewards movement, jumping, jogging and running backwards) and showing the intensities at which each movement was to be performed. Participants completed the circuit until they felt comfortable with the succession and intensity of the movements. They were then weighed in minimal clothing using portable scales (SECA Model 770, Germany) accurate to 0.1 kg. Participants were told to consume fluids ad libitum from a pre-weighed bottle. A simulation of two quarters of a basketball game (Figure 1) was performed. Each circuit commenced at 30 second intervals, as previously described by Scanlan et al. [16]. Each 5-minute block of “game play” contained ten circuits of the BEST. This was followed by three sets of suicide runs (maximal sprint covering a total of 143 m consisting of 4 out and back components from the basketball baseline, the first shuttle covers 5.8 m this increases to 14.0, 22.2, and 28.0 m from the baseline on subsequent shuttles). Upon completion of the third suicide run post-exercise body mass was measured and recorded. Ad libitum fluid intake rate (mL · min^−1^) was calculated and used to determine fluid intakes for each participant during the intervention sessions.

### Intervention testing & sample collection

Following a baseline fasting (venous) blood and saliva sample at 8:00 am, participants were randomly provided with either the PRO/CHO or carbohydrate meal (Table 1). The meals also contained jam (Craig’s Raspberry Fruit Jam, Heinz Wattie’s Ltd, Hastings, New Zealand) and sports drink (Powerade Isotonic, The Coca-Cola Company, Northmead, Australia), however the quantity varied between the two meals. For the PRO/CHO meal, whey protein powder (Horley’s Ice Whey Creamy Vanilla, Nutralac Nutrition Ltd., Mount Eden, New Zealand was ingested to meet the 1 g CHO/kg bm and 1 g PRO/kg bm requirements. As there was more fluid incorporated into the CHO meal due to the greater volume of sports drink consumed, fluid intake was matched in the PRO/CHO meal by giving participants additional water. The final macronutrient composition was 2 g CHO/kg bm for the CHO only meal, and 1 g PRO/kg bm with 1 g CHO/kg bm for the PRO/CHO meal. The amount and timing of the meals was initially based on the protocol and composition of the study by Rowlands and Hopkins [22]. However, unpublished pilot data whereby lower amounts of carbohydrate and protein were provided resulted in participants complaining of hunger during the subsequent exercise session. Therefore the amount of solid food i.e. bread was increased to attenuate hunger. When larger amounts of protein were ingested bloating and nausea were recorded on the GI questionnaire [18], by participants, the timing of ingestion was kept constant during this pilot testing. This meal was consumed within 20 minutes, no participant reported any trouble with consuming the meal in this timeframe. Although the drinks were served in opaque containers participants could determine which trial they were undertaking, however, they were unaware of the hypothesis of the study. They were also not provided with any feedback on their performance measures and it is unlikely that knowing the trial they were performing would have influenced the physiological measures. Participants then rested while anthropometric measures were obtained and used to calculate percentage body fat [23]. Eighty minutes post-meal, participants arrived at the basketball court where a pre-exercise urine sample was obtained. They were provided with water to match their ad libitum fluid intake rate, and a heart rate monitor was fitted (Garmin Forerunner 110, Garmin International Inc., Kansas, USA). Heart rate was automatically transmitted wirelessly to the reciever and recorded every ten seconds. Resting blood lactate and blood glucose concentrations were also obtained. Participants then completed the warm up and free throws as per the familiarisation. An extended “full-game” simulation of four quarters was then performed (Figure 1). Briefly, the exercise protocol was seven blocks (ten circuits per block) of BEST circuits [16] with breaks for free throws and rest periods, with three suicide runs to complete the protocol (Figure 1). Between blocks 4 & 5 there was a 15-minute half time rest period, during which blood glucose and blood lactate concentrations were measured. All other rest periods from blocks 1–7 were 2-minutes duration for either free throws or quarter-breaks. Block 7 was followed by free throws, a 5-minute rest period and three suicide runs separated by 60-seconds rest. Immediately following testing, ear-lobe blood, saliva and urine samples were obtained. At 30-minutes post-exercise, a venous blood sample was obtained. Venous blood, saliva and urine samples were taken again the following morning (referred to as 24-hours) in a fasted state.

**Table 1:** Composition of the CHO and PRO/CHO meals for a 75 kg individual

**3.0a: Composition of the CHO meal for a 75 kg individual**						
**Food item**	**Portion size**	**Energy (kcal)**	**CHO (g)**	**Protein (g)**	**Fat (g)**	**Fibre (g)**
White bread	2 slices	170	36	6	2	1
Jam	60 g	156	42	0	0	0
Sports drink	1290 mL	309	73	0	0	0
**Total**		**635**	**150**	**6**	**2**	**1**
**3.0b: Composition of the PRO/CHO meal for a 75 kg individual**						
**Food item**	**Portion size**	**Energy (kcal)**	**CHO (g)**	**Protein (g)**	**Fat (g)**	**Fibre (g)**
White bread	2 slices	170	36	6	2	1
Jam	40 g	104	28	0	0	0
Protein drink	76 g	292	1	69	0.6	0
Sports drink	190 mL	45	10	0	0	0
Water	1100 mL*	0	0	0	0	0
**Total**		**611**	**75**	**75**	**2.6**	**1**

*600 mL with protein powder + additional 500 mL to equal fluid intake of CHO meal.

### Sample analysis

Venous blood samples were obtained with participants seated in a semi-reclined chair. Samples were collected into heparinized tubes and were centrifuged at 3000 rpm for 15 minutes at 4°C (CR3i, Juan S.A., St. Herblain, France), and separated plasma was stored at −20°C to be later analysed for creatine kinase using a Cobas® C311 Analyser (Roche Diagnostics, Indianapolis, USA). Ear- prick blood samples were analysed for blood lactate (Nova Biomedical, Flintshire, UK), and blood glucose concentrations (Freestyle Optimum, Abbott Diabetes Care, Oxon, UK).

In accordance with the manufacturers instructions, the saliva samples were obtained more than 60 minutes after a meal [24]. Participants were asked to ensure all food was removed from their mouths following the ingestion of the meal and before saliva sampling. Equal amounts of water were ingested in both exercise sessions, with the final drink being consumed at least 10 minutes prior to the saliva sample being obtained [24]. Saliva samples were obtained by asking the participant to passively drool into a small tube until 5 mL of saliva was collected. Saliva was visually inspected for any visible signs of blood contamination [25]. Samples were stored (−20°C) for later analysis of testosterone and cortisol concentrations using a Salimetrics® salivary enzyme immunoassay kit (Salimetrics LLC, State College, Pennsylvania, USA). Urine samples (25 mL) were measured for urine specific gravity on the day of collection using a handheld refractometer (ATAGO, Tokyo, Japan).

### Questionnaires

Participants completed a “Before Meal” GI discomfort questionnaire [18], and then “Before Exercise”, “During Exercise” (15-minute rest period for half-time), and “Following Exercise” (post-suicide runs) GI discomfort and MS questionnaires [19] were completed. A final “Following Day” MS questionnaire was completed at 24-hours. At quarter and half-time breaks, and immediately following suicide runs, RPE was obtained using the Borg scale [20].

### Statistical analysis

All data was analysed using Stata IC 12.0 (StataCorp LP, College Station, Texas, USA). Data was initially tested for normality of distribution using a Shapiro-Wilk test. Where normally distributed, data is presented as mean ± standard deviation (SD). Where not normally distributed, data is presented as mean ± standard error of the mean (SEM) or mean (range). To determine differences between interventions for mean sprint time, jump height, mean and peak heart rate, blood lactate concentration, blood glucose concentration, GI discomfort and MS, a paired t-test was carried out. A Wilcoxon signed-rank test was carried out to determine differences between interventions for mean cortisol concentration, testosterone concentration, creatine kinase concentration, RPE and free throw success rate. A repeated-measures ANOVA with Bonferroni post-hoc test was conducted to establish any time by trial differences. Statistical significance was set at p < 0.05.

## Results

### Heart rate, blood lactate and blood glucose

**Figure 2:**
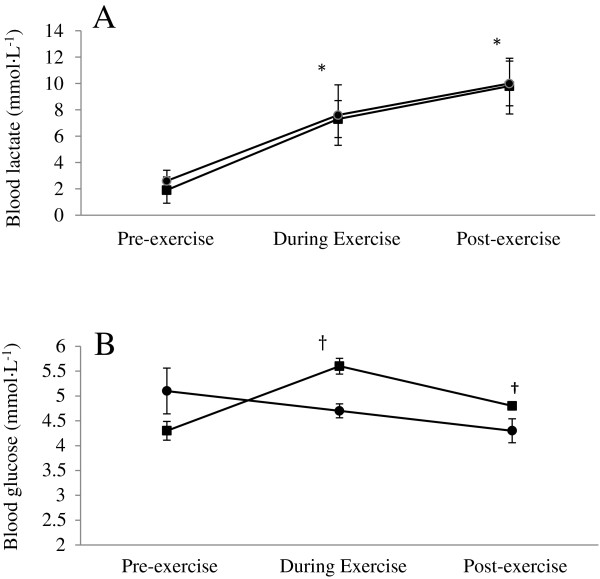
**Blood measures. (A)** blood lactate concentration and **(B)** blood glucose concentration pre-exercise, during exercise, and post-exercise for PRO/CHO = ■ and carbohydrate = ●.

There was no difference between groups for mean heart rate (PRO/CHO: 148 ± 10 beats · min ^−1^; carbohydrate: 149 ± 6 beats · min^−1^; p = 0.506), or peak heart rate (PRO/CHO: 189 ± 7 beats · min ^−1^; carbohydrate: 187 ± 7 beats · min^−1^; p = 0.206). There was a time effect (p < 0.001) for blood lactate (Figure 2A), with increases from before exercise to during exercise (half-time) and again from during exercise to post-exercise, however no trial effect was found (p = 0.441). For blood glucose (Figure 2B), there was no statistical difference at baseline (p = 0.125), however, higher concentrations were seen during and post-exercise for the PRO/CHO trial compared to the carbohydrate trial (p < 0.001 for both time points).

### Performance measures

**Figure 3:**
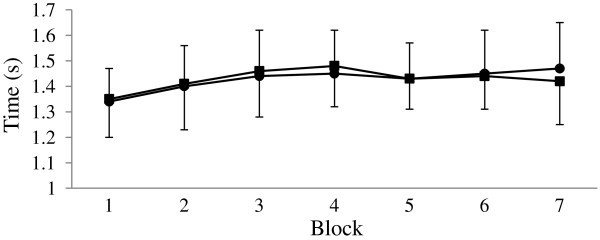
Sprint time (s) for each block of ten consecutive BEST circuits during the exercise protocol for PRO/CHO = ■ and carbohydrate = ● (mean ± SD).

No statistical difference between trials was found at any time point for mean jump height (p > 0.05). A tendency for a faster mean sprint time during the PRO/CHO trial was found during the seventh (final) block of circuits (p = 0.093)(Figure 3). A difference in mean free throw success rate for the first 2 free throw attempts within each set performed in between BEST circuits was found between trials for the fourth quarter (PRO/CHO: 1.6 ± 0.2; carbohydrate: 1.1 ± 0.3; p = 0.027), but did not differ at any other time point (p > 0.05).

### Creatine kinase

A smaller increase in the mean change in CK concentration from baseline (PRO/CHO; 330 ± 81 IU.L^−1^ CHO; 296 ± 88 IU.L^−1^) to post-exercise was found following the PRO/CHO (330 ± 81 to 326 ± 77 IU.L^−1^) compared to the carbohydrate meal (393 ± 81 IU.L^−1^) (p = 0.018) (Table 2). However, there was no statistically significant difference between trials from post-exercise to 24-hours (PRO/CHO 363 ± 79, CHO 394 ± 55 IU.L^−1^, p = 0.401).

**Table 2:** **Mean ± SEM change in creatine kinase (IU · L**^**−1**^**) between baseline and post-exercise, baseline and 24-hours, and post-exercise & 24-hours for PRO/CHO and carbohydrate trials (n = 8)**

	**Change in creatine kinase (IU · L**^**−1**^**)**		
	PRO/CHO	Carbohydrate	P-value
Baseline & post-exercise	56 ± 13	100 ± 10	0.018^†^
Baseline & 24-hours	91 ± 49	97 ± 73	0.866
Post-exercise & 24-hours	37 ± 38	2 ± 63	0.484

^†^Significant difference between trials.

### Testosterone & cortisol

**Figure 4:**
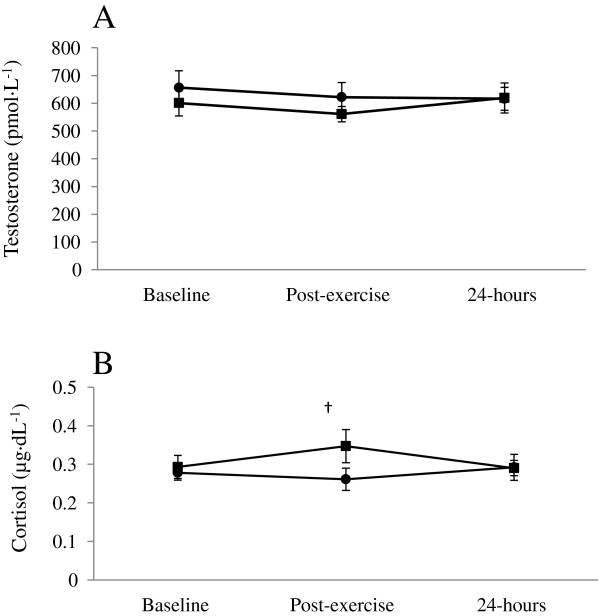
**Salivary hormones. (A)** testosterone and **(B)** cortisol concentration at baseline, post-exercise and 24-hours for PRO/CHO = **■** and carbohydrate = ●.

There was no significant difference between trials for testosterone concentration (Figure 4A). Cortisol concentration was found to be significantly higher for the PRO/CHO trial post-exercise (p = 0.038). However there was no significant difference between trials at baseline or 24-hours (Figure 4B).

### Muscle soreness

There was no significant difference in reported muscle soreness between groups for the upper body, lower body or whole body before exercise, during exercise, post-exercise or at 24-hours (p > 0.05 at all time points). Overall, on the scale from 0 to 8, values ranged from 0–5 during exercise, 0–6 post-exercise and 0–4 at 24 hours for muscle soreness during the PRO/CHO trial, and from 0–6 for all three of these time points during the carbohydrate trial.

### Gastrointestinal discomfort

Absolute values for GI discomfort showed an increase in nausea during exercise (p = 0.007) and post-exercise (p = 0.039). The change in reported severity of symptoms from baseline to during exercise was significantly different between trials for nausea (p = 0.008) and belching (p = 0.017), with the PRO/CHO meal resulting in increases for both symptoms. The change in severity of symptoms from baseline to post-exercise was also significantly different for nausea (p = 0.011) and stomach bloating, which again resulted following the PRO/CHO meal.

### Ratings of perceived exertion

A significant difference in mean RPE between trials was found for quarter 1 (PRO/CHO: 15.8 ± 0.4; carbohydrate: 14.7 ± 0.4 (p = 0.017)) and quarter 4 (PRO/CHO: 17.6 ± 0.6; carbohydrate: 16.6 ± 0.7 (p = 0.017)).

## Discussion

The attenuation of the rise in CK concentration from baseline to post-exercise seen during the PRO/CHO trial, although statistically significant (44 IU^.^L^−1^), appears to have minimal effects, given the lack of any difference in muscle soreness or performance measures such as sprint time and jump height.

The small difference in CK post-exercise between trials is probably not large enough to induce any differences in performance i.e. sprint time or jump height is in agreement with the findings of Valentine et al. [9], who also reported attenuated CK following PRO/CHO consumption during exercise, despite finding no difference in cycling time to exhaustion. Similar findings have also been seen during cross-country running [26].

Although there was a significant attenuation of CK during the PRO/CHO trial and exercise intensity was similar between trials (as indicated by similar lactate and heart rate values between trials), PRO/CHO was not found to affect the changes in muscle soreness compared to carbohydrate alone at any time point. This is potentially due to the small difference in CK not being sufficient to induce any changes in muscle soreness. However, Thompson et al. [19] found poor correlations between changes in muscle soreness and increases in CK levels post-exercise following intermittent shuttle running. Therefore, although muscle soreness did not differentiate between groups in the present study, this perhaps does not appropriately reflect the extent of muscle damage caused throughout the basketball game simulation.

It is possible that during the PRO/CHO trial there was lower muscle and liver glycogen utilisation both at rest and during exercise due to the greater insulin response following the ingestion of protein [27,28] and potentially the greater fatty acid oxidation, thereby attenuating the decrease in blood glucose concentrations towards the end of exercise [22]. Furthermore, gastric emptying is slower with a protein solution in comparison to a glucose beverage [29,30]. This could have lead to a more sustained, gradual release of glucose and protein into the circulation during the present study during the PRO/CHO trial. However, as none of these factors were measured it cannot be determined if any of these had a significant influence.

Cortisol has been shown to increase with heavy exercise [31] especially during intense exercise [32]. Utter, et al. [33], reported that at the end of exercise higher cortisol concentrations were associated with higher blood glucose and lower RPE values. This is in line with the present study and suggests that the blood glucose response could have mediated the cortisol and RPE values towards the end of the exercise session.

Contrary to cortisol concentration, there was no significant difference in testosterone concentration between trials at any time point. These findings are reflective of the previous research by Roberts et al. [12], which is expected given the similarities in study protocol compared with the present study. The results for testosterone suggest that PRO/CHO does not affect the anabolic stimulus to exercise when ingested solely pre-exercise.

The almost 19% increase in nausea from pre-exercise to during exercise during the PRO/CHO trial may have been caused by a decrease in blood flow to the gut upon commencing exercise, insufficient time allowance for protein digestion and absorption pre-exercise, or unfamiliarity to the amount of protein (1 g · kg^−1^ body mass) ingested for the majority of participants. The unfamiliarity of the protein dose may also be the reason for the increased cortisol levels found post-exercise following the PRO/CHO meal in response to the GI distress experienced by participants during exercise [34]. Although the main issues reported in our pilot data were hunger with nobody reporting any GI distress until the main trials it is possible that the main trials were of a higher intensity (however, as heart rate was not measured in the pilot testing this is speculative) combined with the longer duration resulted in these negative effects only appearing at this timepoint.

These elevations in GI distress may be one of the reasons for the significantly higher RPE values reported following the first quarter and the fourth quarter (p = 0.017). However, for both time points the increase in mean RPE was by one point for the PRO/CHO trial. This small increase is unlikely to indicate any real or substantial increase in how hard participants felt they were working.

Furthermore, RPE is a subjective measure of the effort of exertion being felt, and it could be that the increase in nausea reported by participants during the PRO/CHO trial influenced their ratings of RPE, rather than because they felt it was harder to perform the movements during the exercise protocol. Alternatively pre-conceived ideas around carbohydrate and protein intake before exercise may have influenced this subjective measure whereby the carbohydrate trial had a lower RPE because the athletes believed that carbohydrate intake would allow then to perform better and so the exercise seemed less difficult. However, knowledge and beliefs concerning macronutrient intakes was not measured therefore conclusions about this cannot be determined.

Future studies need to adjust meal composition and timing in order to decrease nausea, following which it could be revealed whether the pre-exercise protein ingestion or the nausea is responsible for the differences in cortisol and RPE values found post-exercise in the present study. More long term studies where participants are acclimatised to increased protein intakes before exercise may yield more positive results. However, the major limitation of the present study was the differences in the appearance of the carbohydrate sports drink and the protein drink, future studies may wish to provide drinks similar in appearance and taste.

In order to decrease GI distress, training the gut to cope with larger amounts of protein during times where muscle damage needs to be attenuated (such as during tournaments and times of increased training load) may be required so that pre-exercise absorption rate may be increased. This theory is similar to the proposals of Jeukendrup [35] for carbohydrate intake for endurance athletes, whereby a gradual increase in carbohydrate allows the gut to adapt and tolerate greater quantities during exercise. Murray [36] also suggests that practising fluid and nutrient intake during training is important for optimal gastrointestinal function.

## Conclusion

In conclusion, for well-trained basketball players, pre-exercise PRO/CHO co-ingestion appears to have limited effects on performance and muscle damage. It may maintain free throw shooting accuracy during the final minutes of game play, whilst maintaining sprint speed and jumping ability in comparison to carbohydrate alone.

Although findings are encouraging, unfamiliarity to the dosage of protein provided in the PRO/CHO meal may have caused nausea to increase during exercise, and this may have limited the ability to see an improvement in more performance measures. Therefore, it may mean that athletes need to vary the amount and timing of protein intake in training to discover a protocol which does not cause them GI distress. They may then gradually increase protein amounts, so as the gut can become accustomed to higher intakes and the beneficial effects of pre-exercise PRO/CHO co-ingestion can occur without GI comfort being compromised.

## Abbreviations

PRO/CHO: Protein and carbohydrate; CK: Creatine kinase; BEST: Basketball exercise simulation test; CHO: Carbohydrate; GI: Gastrointestinal; MS: Muscle soreness; RPE: Rating of perceived exertion; Bm: Body mass.

## Competing interests

None of the authors have any conflicting interests.

## Authors' contributions

HG, KB made substantial contributions to the study design, statistical analysis, data collection and interpretation and manuscript preparation. TL made substantial contributions to the study design, statistical analysis, data interpretation and manuscript preparation. AH statistical analysis, data collection and interpretation and manuscript preparation. All authors read and approved the final manuscript.
